# Perceived Barriers and Use of Evidence-Based Practices for Adolescent HPV Vaccination among East Texas Providers

**DOI:** 10.3390/vaccines11040728

**Published:** 2023-03-25

**Authors:** Sarah Kim, Kelvin Zhou, Susan Parker, Kimberly N. Kline, Jane R. Montealegre, Lindy U. McGee

**Affiliations:** 1 Department of Medical Education, Baylor College of Medicine, Houston, TX 77030, USA; 2 Dan L. Duncan Comprehensive Cancer Center, Baylor College of Medicine, Houston, TX 77030, USA; 3 Department of Communication, The University of Texas at San Antonio, San Antonio, TX 78249, USA; 4 Department of Pediatrics, Baylor College of Medicine, Houston, TX 77030, USA

**Keywords:** HPV vaccine, rural health care, Texas, barriers

## Abstract

Adolescents living in rural areas are less likely to be up to date on the human papillomavirus (HPV) vaccine, which can prevent cervical cancer. We administered a telephone survey to 27 clinics in rural East Texas to assess perceived barriers to HPV vaccination and current use of evidence-based interventions to promote HPV vaccination. Perceived barriers were assessed using a 5-point Likert scale and clinical implementation of evidence-based practices was determined. Findings are reported using descriptive statistics. The most commonly reported barriers were missed vaccination opportunities due to the pandemic (66.7%), followed by vaccine hesitancy due to the pandemic (44.4%) and due to the HPV vaccine specifically (33.3%). Fewer than a third of clinics reported using the evidence-based strategies of use of a “refusal to vaccinate” form (29.6%), having an identified HPV vaccine champion (29.6%), and recommending the HPV vaccine at age 9 (22.2%). While many clinics surveyed currently implement evidence-based practices to promote HPV vaccination, there is a need and desire for additional HPV vaccination interventions in East Texas clinics.

## 1. Introduction

Each year in the U.S., an estimated 36,500 people are diagnosed with new cases of human papillomavirus (HPV)-attributed cancer [1]. Approximately 94% of those cancers can be prevented by the 9-valent HPV vaccine [1]. Despite this, U.S. HPV vaccination rates remain lower than the rates of other vaccines recommended during adolescence [2]. Texas ranks 47 out of the 50 U.S. states for HPV up to date (UTD) rates, with only 51.5% of adolescents aged 13 to 17 years UTD on the vaccine [2]. Disparities are present between rural and urban vaccination rates, with adolescents living in rural non–metropolitan statistical areas (MSA) having the lowest HPV vaccine UTD rates [2,3].

There are numerous barriers associated with adolescent vaccine delivery and uptake. Parental concerns about vaccine safety, spurred by online misinformation [4], may dissuade parents from vaccinating their children [5,6,7]. There is evidence that this vaccine hesitancy has increased with online misinformation spread during the coronavirus disease 2019 (COVID-19) pandemic [4,8]. In the U.S., childhood and adolescent vaccines are predominantly delivered in a health-care setting. Immunization therefore faces challenges related to health-care access, including transportation issues, time constraints, and cost of stocking vaccine [6]. Furthermore, while frequent visits to a health-care provider are the norm in childhood, adolescents infrequently visit a health-care provider [9]. This has also worsened with the pandemic, when many clinics struggled to provide routine care [10]. In states without effective centralized immunization record systems, such as Texas, lack of access to vaccine records also presents a barrier to UTD immunization [11].

Other barriers to UTD HPV vaccination rates are more specific to the HPV vaccine. These include parent factors, such as the belief that vaccination may lead to sexual promiscuity and lack of awareness of HPV disease or risk of infection [7]. Also, due to online misinformation about the HPV vaccine specifically, parents who are not hesitant about other vaccines have refused the HPV vaccine, citing safety concerns [5]. At the provider level, lack of strong provider recommendation for the vaccine has consistently been found to be a leading reason for low HPV immunization rates [12].

Multiple clinical practices and interventions in the health-care setting have been shown to be effective in increasing HPV vaccine delivery and uptake. These are most effective when used together [13,14]. These include strategies targeting providers, such as assessment and feedback and communication training, and strategies to educate patients and parents [13,14]. Routine use of good clinical practices is recommended to decrease missed opportunities for vaccination: nurse visits for vaccination, standing delegated orders for vaccination, vaccination at sick visits if the patient qualifies clinically, electronic medical record (EMR) prompts, and review of immunization registry records [15]. Additionally, the American Academy of Pediatrics and the American Cancer Society recommend starting the HPV vaccine at age 9 years [16,17] as an established strategy to increase vaccine UTD rates by age 13 years [18].

The challenges associated with HPV vaccine delivery are amplified in rural areas of the country. A recent study by Pruitt et. al. found that adolescents in rural areas were more likely to have missed HPV vaccine opportunities [19]. Interviews of stakeholders in rural North and South Carolina revealed individual-level concerns related to lack of knowledge and spread of misinformation about the HPV vaccine: provider-level barriers of lack of pediatric providers, irregular well child visits, difficulty obtaining and storing the vaccine, and lack of strong recommendations from providers; and system-level barriers such as lack of a school mandate for the vaccine [20]. Other studies suggest that rural disparities may be explained by shortages of primary care providers and decreased public knowledge about the HPV vaccine [3,21,22].

In Texas specifically, HPV vaccine UTD rates are higher in the urban areas of Houston (62.2%) (population density 3598 people per square mile) and Bexar County (59.7%) (1620 people per square mile) than in the rest of the state (50.0%) [2]. Unfortunately, rural East Texas counties have some of the highest rates of HPV-associated cancers [23]. Targeted interventions to improve HPV vaccine uptake are critical to mitigate future cancer burden and decrease urban–rural disparities. As a first step, we conducted a needs assessment to understand current clinical practices regarding HPV vaccination in rural East Texas primary health-care settings, assessing health-care providers’ perceived barriers to HPV vaccination, as well as current strategies in place to address those barriers.

## 2. Materials and Methods

The Institutional Review Board of Baylor College of Medicine and Affiliated Hospitals reviewed and approved this study (protocol H-44624). Clinics were geographically defined as those in the Public Health Region 4/5 as designated by the Texas Department of State Health Services covering 38 rural East Texas counties. A list of clinics within each county was generated by referencing the database of clinics participating in the Texas Vaccines for Children (VFC) program, a federal program that provides vaccines free of charge to qualifying children, as well as the database of clinics participating in a major Texas health plan. Additional clinics were included by reference from participants who completed the survey. Clinics were reached by telephone by a trained interviewer. The interviewer requested scheduling an interview with the clinic staff member most knowledgeable about vaccine supply and barriers in the clinics. An interviewer-administered survey was conducted by telephone on the agreed date and time. Study data were collected and managed using REDCap electronic data capture tools [24,25] hosted at Baylor College of Medicine.

The survey instrument consisted of a 50-item questionnaire developed by the research team based on a thorough review of the literature on HPV vaccine-promoting clinical practices [26,27], including Centers for Disease Control and Prevention recommendations [15] (see Supplementary Material Supplement 1). Participants were asked for their role in the clinic (nurse, medical assistant, physician, administrative/management, other), as well as questions to determine clinic type (federally qualified health centers, private practice, hospital-based clinics, public health department-operated clinics, other) and setting (rural, suburban, urban). Patient demographics were assessed by asking participants the estimated percentage of patients from each racial/ethnic group (Latino, Hispanic, non-Hispanic Black, non-Hispanic White, non-Hispanic other) and insurance status (private, Medicaid, uninsured). Participants were asked if their clinic participated in the VFC program and whether they had privately stocked HPV vaccine (i.e., not provided by the VFC program).

Perceived barriers to HPV vaccination were assessed using a 5-point Likert scale (strongly disagree, disagree, neutral, agree, strongly agree). Implementation of clinical practices known to increase HPV vaccination rates was determined by listing strategies and asking participants to answer yes, no, or unsure. The presence and capabilities of an EMR were determined, as well as whether the clinic was currently participating in HPV vaccine initiatives or would be interested in joining one. Additional questions were added concerning the impact of the COVID-19 pandemic on vaccination. At the end of the survey, participants were asked the open-ended question “Which strategies has your clinic found to be most helpful?” Responses were recorded verbatim.

All questions were based on participant knowledge and recall. Each phone survey took approximately 15 min to complete, and participants received a $10 gift card in appreciation of their time. In addition to survey data, characteristics of counties of participating clinics were assessed from public data sources: county population density [28] and percentage of population without health-care insurance [29].

For the analysis, Likert scale responses to perceived barriers were collapsed into three categories (disagree, neutral, agree). Descriptive statistics were used to summarize perceived barriers and implementation of clinical practices. Open-ended responses were coded by the senior author and organized into themes.

## 3. Results

Between July and September 2022, the research team contacted 161 clinics by telephone and the phone survey was completed by representatives of 27 clinics (response rate = 16.8%). Interviewees consisted of nursing staff, medical assistants, and management personnel (data not shown). The clinics were located in 18 East Texas counties with population densities ranging from 13.1 people per square mile to 454.5 people per square mile (Table 1). All clinics except for one were located in counties with >10% uninsured rates for those aged 18 years and under. Most (81.5%) clinics were located in counties designated as primary care health professional shortage areas [30]. The clinics varied from federally qualified health centers (33.3%), private practice (44.4%), hospital-based clinics (11.1%), public health department-operated clinics (7.4%), and other (1). (Table 1) Most described their practice setting as rural (81.5%) versus suburban (18.5%). All clinics provided routine childhood and adolescent vaccines, and 22.2% also provided adult vaccines. All clinics participated in the VFC program and 63% of clinics also purchased private stock HPV vaccines. Most practices were small, with 2–5 providers (74.1%). In the majority of clinics (59.3%), adolescent patients are seen by family practice-trained providers, compared to pediatric-trained providers (25.9%) or both types (14.8%). The clinics served a racially and ethnically diverse patient population, with 55.6% of clinics serving at least 25% Hispanic/Latino patients and 63% serving at least 25% Black patients. Most of the clinics (77.8%) had a patient population of at least 50% who were insured by Medicaid.

The most prevalent perceived barrier was missed opportunities for vaccination because of the COVID-19 pandemic (66.7%). There was also concern about increased general vaccine hesitancy as a result of the pandemic, with 44.4% of respondents in agreement with the statement “Parental hesitancy regarding vaccines has increased since the COVID pandemic” and 7.4% neutral. Respondents also recognized that there is hesitancy around the HPV vaccine specifically, with only 14.8% of respondents disagreeing with the statement “Parental vaccine hesitancy regarding HPV vaccine specifically is a significant problem in this practice”. Other barriers identified were lack of vaccine records (29.6% agree), language and cultural barriers (14.8% agree), parental hesitancy regarding all childhood vaccines (14.8% agree), and cost of vaccine (7.4% agree).

**Figure 1:**
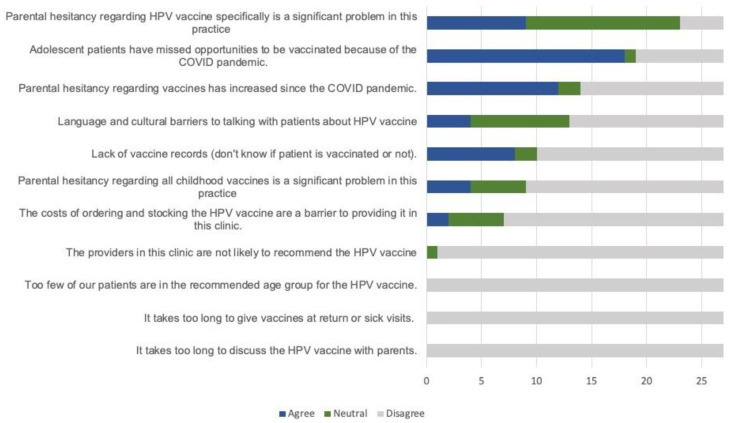
Perceived barriers to HPV vaccination.

The following were not identified as barriers in any clinic: it takes too long to discuss the HPV vaccine with patients, it takes too long to give the vaccines at return or sick visits, and too few of our patients are in the recommended age-group for the HPV vaccine. (Figure 1) One clinic responded neutral to the statement “The providers in this clinic are not likely to recommend the HPV vaccine,” and none agreed with the statement.

In regard to HPV vaccine-promoting clinical practices, all participants reported that their clinic providers recommend the HPV vaccine at age 11 bundled with other vaccines and that educational materials are provided to parents/caregivers (Table 2). A majority also identified using the following strategies to increase vaccination rates: checking state registry for immunization records (92.6%), having staff undergo training on HPV vaccine communication (81.5%), regularly monitoring HPV vaccination rates of the patient population (63.0%), and using standing delegated orders for vaccines and/or giving vaccines prior to provider interaction (59.3%). Most clinics offered flexible vaccination opportunities, with 96.3% offering vaccines on weekdays before or after school and 55.6% on weekends. Most provided immunization-only visits (88.9%) and offered the HPV vaccine at acute visits if the patient was well enough (77.8%). The majority reported the use of systems such as electronic medical record alerts to remind providers if patients were due for vaccination (74.1%) and to remind parents for first (66.7%) and subsequent doses (70.4%). The least implemented strategies included having parents sign a “refusal to vaccinate” form if they declined (33.3%), having an identified “HPV vaccine champion” in the clinic (37.0%), and recommending the HPV vaccine at age 9 (37.0%).

When asked the open-ended question “Which strategies has your clinic found to be most helpful?”, strategies involving provider communication were identified most often. (Table 3) Respondents especially recognized reassuring communication and answering parents’ questions and concerns as important strategies to increase vaccination rates, with one respondent stating “Face to face is better than written communication.” There was also recognition that the entire staff needs training on strong vaccine recommendations. Other themes identified for strategies were parent education and system-based strategies such as scheduling follow-up appointments at the time of vaccination.

## 4. Discussion

Several findings emerged from the survey data of HPV vaccination barriers and strategies in place at East Texas clinics. The first was the impact of parent vaccine hesitancy as a barrier to immunization. The participating clinics overwhelmingly identified that their providers do recommend the HPV vaccine and that they offer the vaccine at both sick and well adolescent visits. Additionally, most clinic providers (81.5%) had undergone some type of training on HPV vaccine communication, in accordance with current recommendations [15]. Despite these known effective strategies for countering hesitancy, survey respondents still recognized parental vaccine hesitancy as a barrier to HPV vaccination in their clinics. This is consistent with prior surveys and interviews of rural health providers and stakeholders who identified vaccine misinformation causing hesitancy as a major barrier in rural communities [20,26]. Respondents asserted that face-to-face communication between the provider and parent and addressing any concerns remain the most effective strategies to fight parental hesitancy. Clinics also used written educational materials to supplement those conversations. Although vaccine hesitancy is not unique to rural communities, several factors may make it more of a challenge in those communities. A study by Mohammed et al. found that compared to urban counterparts, rural adults are less likely to have heard of HPV or the HPV vaccine and less likely to believe that HPV can cause cancer [22]. Decreased access to a medical home and more infrequent visits for adolescents in rural settings [31] give providers fewer opportunities for education and for building a trusting relationship with parents [3].

Another major finding was in regard to the perceived impact of the COVID-19 pandemic on HPV immunization rates. This concern mirrors findings in other parts of the U.S., which have shown reduced levels of HPV vaccination during the pandemic, especially early in the pandemic [32,33]. Similarly to national trends, East Texas clinics cited missed opportunities because of the pandemic due to decreased number of visits as well as increased vaccine hesitancy in response to the pandemic [4] as major barriers to UTD vaccination. Future studies are needed to determine if this impact is greater in rural communities.

Finally, we found that fewer than one-quarter of the East Texas practices surveyed started recommending HPV vaccination at age 9. Incorporation of this strategy could help to address the concern about missed opportunities, through introduction at an age where children are more likely to come in for annual well child exams. Earlier introduction also allows for more time for provider communication to counter vaccine hesitancy. Studies report increased parental vaccine uptake by age 13 when the discussion begins at earlier visits [16,18,34].

This survey serves as an initial look into perceived barriers to HPV vaccination in rural East Texas and categorizes strategies clinics are currently using to address those barriers. One limitation of this study is that by initially calling providers who are enrolled in the VFC program, we may have selected for clinics with better HPV vaccine policies and for those who are more likely to stock the vaccine, which has been shown to be a barrier in other studies [20]. We attempted to mitigate that limitation by asking survey respondents for other clinics in their area. We also had a low response rate for the clinics, which further limits the generalizability of the data. Additionally, perceived barriers and clinical practices were based on respondent knowledge and recall and did not involve verification by review of clinical records. This study did not assess the barriers perceived by the patients or their parents or compare rural to urban clinics, and is therefore a descriptive analysis, not a comparative one. The survey focused on clinic-level strategies and therefore did not assess for the strategy of school- and pharmacy-based vaccination programs, which have been proposed to facilitate vaccination in the wake of the pandemic [35,36]. A strength of the study was that we were able to obtain in-depth information from the clinics who did respond, and we had a diversity of provider types who responded. Our findings should thus be viewed as exploratory. Future research is needed to better understand parental hesitancy and whether it is increased in this rural area compared to urban areas. Future interventions in the region should target how clinics address vaccine hesitancy and considering initiation at age 9 as a strategy to allow for more visits for communication, while allowing for vaccine completion by age 13.

## 5. Conclusions

While many clinics surveyed currently implement evidence-based practices to promote HPV vaccination, there is a need and desire for additional HPV vaccination strategies in rural East Texas clinics. Increased vaccine coverage for rural populations should address barriers experienced during the COVID-19 pandemic and communication to vaccine-hesitant parents.

## Figures and Tables

**Table 1:** Clinic characteristics and patient demographics.

Clinic Characteristics		*n* (%) Total *n* = 27
Practice type	Federally qualified health center *	9 (33.3%)
	Hospital-based clinic **	3 (11.1%)
	Private practice ***	12 (44.4%)
	Public health department-operated clinic	2 (7.4%)
	Other	1 (3.7%)
Setting of clinic	Urban	0 (0%)
	Suburban	5 (18.5%)
	Rural	22 (81.5%)
Population density of county (persons per square mile)	≤20	3 (11.1%)
	21–50	8 (29.6%)
	51–100	7 (25.9%)
	101–500	9 (33.3%)
Uninsured population in county, ages 18 and younger	<10%	1 (3.7%)
	10–11.9%	12 (44.4%)
	12–13.9%	10 (37.0%)
	14–15.9%	4 (14.8%)
Clinic in a primary care shortage area	Yes	22 (81.5%)
	No	5 (18.5%)
Vaccines provided to	Child and adolescent patients	27 (100%)
	Adult patients	6 (22.2%)
Participation in Vaccines for Children (VFC) program	Yes	27 (100%)
	No	0 (0%)
Purchase private stock vaccine	Yes	17 (63.0%)
	No	10 (37.0%)
Number of providers in clinic	1	0 (0%)
	2–5	20 (74.1%)
	6–15	6 (22.2%)
	16+	1 (3.7%)
Specialty of providers seeing pediatric patients	Family practice/internal medicine only	16 (59.3%)
	Pediatrics only	7 (25.9%)
	Both provider types in clinic	4 (14.8%)
Race breakdown of patients	Hispanic/Latino 0–25% 26–50% 51–75% 76–100%	12 (44.4%) 7 (25.9%) 7 (25.9%) 1 (3.7%)
	Non-Hispanic Black 0–25% 26–50% 51–75% 76–100%	10 (37.0%) 16 (59.3%) 1 (3.7%) 0 (0%)
	Non-Hispanic White 0–25% 26–50% 51–75% 76–100%	10 (37.0%) 7 (25.9%) 10 (37.0%) 0 (0%)
	Other **** 0–25% 26–50% 51–75% 76–100%	26 (96.3) 0 (0%) 0 (0%) 0 (0%)
Insurance status of patients	Private 0–25% 26–50% 51–75% 76–100%	17 (63.0%) 8 (29.6%) 2 (7.4%) 0 (0%)
	Medicaid 0–25% 26–50% 51–75% 76–100%	0 (0%) 6 (22.2%) 9 (33.3%) 12 (44.4%)
	Uninsured **** 0–25% 26–50% 51–75% 76–100%	22 (81.5%) 3 (11.1%) 1 (3.7%) 0 (0%)

* Includes community, migrant, rural, or Indian health center; ** includes university clinic or residency teaching practice; *** includes solo, group practice, or HMO; **** had one “don’t know” response.

**Table 2:** Strategies clinics use to increase HPV vaccination rates.

Strategy		n (%) Total *n* = 27
Clinic has an identified “HPV vaccine champion” whose job includes monitoring HPV vaccination rates and activities.	Yes	8 (29.6%)
	No	17 (63.0%)
	Unsure	2 (7.4%)
Staff regularly monitors and reviews HPV vaccination rates of our patient population.	Yes	17 (63.0%)
	No	10 (37.0%)
	Unsure	0 (0%)
Staff has undergone training on HPV vaccine communication.	Yes	22 (81.4%)
	No	4 (14.8%)
	Unsure	1 (3.7%)
Start recommending the HPV vaccine at age 9.	Yes	6 (22.2%)
	No	17 (63%)
	Unsure	4 (14.8%)
Recommend the HPV vaccine at age 11, bundled with other vaccines.	Yes	27 (100%)
	No	0 (0%)
	Unsure	0 (0%)
Parents sign a “refusal to vaccinate” form if they refuse the HPV vaccine.	Yes	8 (29.6%)
	No	18 (66.7%)
	Unsure	1 (3.7%)
Provide educational material on HPV vaccine for parents/patients.	Yes	27 (100%)
	No	0 (0%)
	Unsure	0 (0%)
Provide immunization-only visits/nurse visits for immunizations.	Yes	24 (88.9%)
	No	2 (7.4%)
	Unsure	1 (3.7%)
Use standing delegated orders for vaccines and/or gives vaccine prior to provider interaction.	Yes	16 (59.3%)
	No	10 (37.0%)
	Unsure	1 (3.7%)
Offer the HPV vaccine at acute care/sick visits if patient is well enough to vaccinate (not only at well child checks).	Yes	21 (77.8%)
	No	5 (18.5%)
	Unsure	1 (3.7%)
Provide immunization appointment times before and after school on weekdays.	Yes	26 (96.3%)
	No	1 (3.7%)
	Unsure	0 (0%)
Provide vaccine appointment times on weekends.	Yes	15 (55.6%)
	No	12 (44.4%)
	Unsure	0 (0%)
System to remind provider if patient is due for vaccine (such as electronic medical record alerts).	Yes	20 (74.1%)
	No	5 (18.5%)
	Unsure	2 (7.4%)
System to remind parents of first dose (such as electronic alert, call, postcard, text).	Yes	18 (66.7%)
	No	7 (25.9%)
	Unsure	2 (7.4%)
System to remind parents when second and/or third dose is due (such as electronic alert, call, postcard, text)	Yes	19 (70.4%)
	No	6 (22.2%)
	Unsure	2 (7.4%)
Check state registry (ImmTrac2) for immunization records if they are incomplete before the visit or at the time of the visit.	Yes	25 (92.6%)
	No	1 (3.7%)
	Unsure	1 (3.7%)

**Table 3:** Reported strategies perceived to be most helpful for HPV vaccination.

Theme	Quotes
Provider Communication	“One-on-one education during visits for parents and handouts so they can call about it later. Being available to patients to answer questions” (Clinic Coordinator)
	“Talking thoroughly with patients; explaining even if they agree with a procedure, medication, etc. so that everyone is on the same page moving forward” (Family Nurse Practitioner)
	“Talking with parents when kids come in for well-checks. Face-to-face is better than written communication” (Triage Nurse)
	“Talking with parents if they have any concerns” (Office Manager)
	“Talking it through with parents” (Medical Assistant)
	“Reassuring parents” (Nurse Practitioner)
	“Making sure staff is on same page when it comes to vaccination recommendations” (Clinic Manager)
	“Bundling vaccines, advertising throughout clinic” (Care Coordinator)
Parent Education	“Education, providing different resources” (Medical Assistant)
	“Educational material to parents” (Site Manager)
	“Education for parents and patients” (Certified Nurse Assistant)
System-Based	“Scheduling 2nd and 3rd when 1st dose is administered” (Nurse Manager)
	“Providing information and directions for follow-up” (Clinic Coordinator)
	“Immtrac, if able to find one for the patient” (Family Nurse Practitioner)
	“Getting them [adolescent patients] while they’re here [during well-child visits]” (Licensed Vocational Nurse)
	“Being a resource for underserved families (uninsured)” (Physician Assistant)

## Data Availability

The data that support the findings of this study are available from the corresponding author upon reasonable request.

## Supplementary Materials

The following supporting information can be downloaded at https://www.mdpi.com/article/10.3390/vaccines11040728/s1. Supplement 1: East Texas Clinic Survey
