# Supplementary material for: Perceived Barriers and Use of Evidence-Based Practices for Adolescent HPV Vaccination among East Texas Providers
Source: Vaccines (Basel). 2023 Mar 25;11(4):728. doi: 10.3390/vaccines11040728 (PMC10146224; doi:10.3390/vaccines11040728)
Supplement: Supplementary file 1 [file vaccines-11-00728-s001.zip › vaccines-2259261-supplementary.pdf]

My First Instrument

Record ID ________________________________

Clinic Name ________________________________

Interviewee ________________________________

HELLO, I am calling from Baylor College of Medicine. My name is ____________ .

Does your practice or clinic ever provide outpatient services to teens aged 11-18?
(IF NO --- Unfortunately, your clinic does not qualify for this survey. Thank you very much for your time. Have a lovely day. If YES, continue with survey)

◯ Yes
◯ No
◯ Don't know / not sure

We are doing a research survey about the barriers to HPV vaccination at health care facilities in East Texas. To do the survey, I will need to speak to the medical provider or staff member who knows the most about HPV vaccine supply and barriers to use in your clinic. As an incentive, the person who completes the phone survey will receive a $10 Amazon gift card.

Is that person available to speak now?

If not, do you have an email address for them so I can reach out to schedule a call?

(Phone surveyor will send an email to the provider / staff member with consent information to review and tables for reference during survey)

Have you had an opportunity to review the consent document sent by email and do you agree to proceed?

(if no, then end survey)

As a reminder, your responses will remain confidential and will be de-identified in any publications.

◯ Yes
◯ No

What is your name? ________________________________

What is your position in the clinic? ________________________________

1. Which of the following BEST describes your practice or clinic?
(Read all; check only one representing the most specific description)

◯ Federally-qualified health center including community, migrant, rural, or Indian health center;
◯ Hospital-based clinic, including university clinic or residency teaching practice;
◯ Private practice, including solo, group practice, or HMO;
◯ Public health department-operated clinic; What type ____________________?
◯ Military clinic
◯ Other ____________________

Clarification

____

2. Which best describes the setting of your practice or clinic?

◯ Urban
◯ Suburban
◯ Rural

3. Do you provide the following in-stock vaccines?
[check all that apply]
((vaccines that they currently have in their refrigerator and can give to patients))

☐ Recommended vaccines for infants and children under the age of 5 such as polio, MMR, and prevnar (pneumococcal)
☐ HPV vaccine
☐ Oher vaccines for adolescents such as meningitis and T-DAP vaccines
☐ Flu shots
☐ Vaccines for adults such as the shingles vaccine
☐ COVID vaccines for 5-11 year olds
☐ COVID vaccine for 12 and up
☐ We do not provide any vaccines for patients in our clinic (if checked this, then skip to question #6)

4. Does your practice or clinic participate in the state supplied vaccine program for HPV vaccine? This includes the federal Vaccines for Children Program also called VFC.
(*If unsure... PROBE: That is, do you give state-supplied vaccines to your uninsured, underinsured, or publicly insured patients?)

◯ Yes
◯ No
◯ Enrollment in program is currently in process

5. Does your practice or clinic purchase HPV vaccines privately (also called "private stock" or vaccines for those with private insurance)?

◯ Yes
◯ No
◯ Enrollment in program is currently in process

6. Do you ever refer patients to other places for the HPV vaccine?

◯ Yes
◯ No
◯ Don't know / not sure

6a. If yes, then where

____

7. On a scale of 1-5 with 1 being strongly disagree and 5 being strongly agree, how much do you agree with the following statements regarding barriers to your practice or clinic with respect to the HPV vaccine?

	Strongly disagree	Disagree	Neutral	Agree	Strongly agree
The costs of ordering and stocking the HPV vaccine are a barrier to providing it in this clinic	○	○	○	○	○
Too few of our patients are in the recommended age group for the HPV vaccine	○	○	○	○	○
Parental hesitancy regarding all childhood vaccines is a significant problem in this practice	○	○	○	○	○
Parental hesitancy regarding HPV vaccine specifically is a significant problem in this practice	○	○	○	○	○
Parental hesitancy regarding vaccines has increased since the COVID pandemic	○	○	○	○	○
The providers in this clinic are not likely to recommend the HPV vaccine	○	○	○	○	○
It takes too long to discuss the HPV vaccine with parents	○	○	○	○	○
It takes too long to give vaccines at return or sick visits	○	○	○	○	○
Language and cultural barriers to talking with patients about HPV vaccine	○	○	○	○	○
Adolescent patients have missed opportunities to be vaccinated because of the COVID pandemic	○	○	○	○	○
Lack of vaccine records (don't know if patient is vaccinated or not)	○	○	○	○	○

Any other barriers at your clinic not covered above? ____________________________

8. What strategies do you use to increase HPV vaccination rates in your clinic?

	YES (Strategy in place)	NO (Strategy not in place)	I DON'T KNOW (Unsure if strategy in place)
Clinic has an identified "HPV vaccine champion" whose job includes monitoring HPV vaccination rates and activities	○	○	○
Provide immunization-only visits / nurse visits for immunizations	○	○	○
Provide immunization appointment times before and after school on weekdays	○	○	○
Provide vaccine appointment times on weekends	○	○	○
Offer the HPV vaccine at acute care / sick visits if patient is well enough to vaccinate (not only at well child checks)	○	○	○
Start recommending the HPV vaccine at age 9	○	○	○
Recommend the HPV vaccine at age 11, bundled with other vaccines	○	○	○
System to remind provider if patient is due for vaccine (such as electronic medical record alerts)	○	○	○
Parents sign a "refusal to vaccinate" form if they refuse the HPV vaccine	○	○	○
Check state registry (ImmTrac) for immunization records if they are incomplete before the visit or at the time of the visit	○	○	○
System to remind parents of first dose (such as electronic alert, call, postcard, text)	○	○	○
System to remind parents when second and /or third dose is due (such as electronic alert, call, postcard, text)	○	○	○
Staff regularly monitors and reviews HPV vaccination rates of our patient population	○	○	○

Use standing delegated orders for vaccines and/or gives vaccine prior to provider interaction	◯	◯	◯
Staff has undergone training on HPV vaccine communication	◯	◯	◯
Provide educational material on HPV vaccine for parents / patients	◯	◯	◯

Other strategy? (please provide)

____

Which strategies has your clinic found to be most helpful? ________________________________

If you have a system to reminder providers and / or parents, what system do you use? ________________________________

If staff has undergone training, which staff and what training? ________________________________

If provide educational material, what material? ________________________________

We are almost done now.
I just have a few more questions about your practice and the people that your practice or clinic sees.

9. About how many providers (physician, nurse practitioner, physician assistants) are in this main primary care practice location who see patients ages 9-17 years?

◯ 1
◯ 2-5
◯ 6-15
◯ 16+

10. What is the specialty of the physicians/ NPs / PAs who see your patients ages 9-17 (check all that apply)?

◯ Pediatrics
◯ Family practice
◯ Internal medicine/pediatrics
◯ Adolescent Medicine
◯ Ob/Gyn
◯ Other: __________________

10a. Other ________________________________

11. What type of medical record system does your main primary care practice use? (Don't need to read options)

◯ Paper charts
◯ Partial electronic medical records (e.g., lab results available electronically, but patient history on paper)
◯ In transition from paper to full electronic medical records
◯ Full electronic medical records

12. On average, estimate how many 9-17 year old patients does your practice or clinic see in a typical day?

____

13. Roughly what percentage of your patients are in the following ethnic/racial group?

If respondent hesitates, then suggest
"Would you say... 0%, 1-9%, 10-24%, 25-49%, 50-74%, 75-100%, don't know"

Hispanic or Latino?

____

Black or African-American?

____

Caucasian?

____

Other?

____

14. Roughly what percentage of your pediatric patients are insured by the following?

If respondent hesitates, then suggest
"Would you say... 0%, 1-9%, 10-24%, 25-49%, 50-74%, 75-100%, don't know"

Have private insurance?

____

Are insured by Medicaid?

____

Are uninsured?

____

15. Is your clinic equipped with wifi internet access?

◯ Yes
◯ No

16. Is your clinic currently participating in any HPV initiatives or projects?

◯ Yes
◯ No
◯ Don't know / not sure

16a. What HPV initiatives or projects?

____

19. If an HPV program is initiated in your county, would you be interested in participating?

◯ Yes
◯ No
◯ Don't know / not sure

20. Do you know the HPV vaccine two-dose completion rates are in your practice?
If so, how do you measure it (ie 15 year old, 13-17 year old)? please provide the number:
If not, please estimate the completion rate for 13-17 year olds:

____

21. Is there anyone else / any other clinics in your area that you think we should consider talking to for this survey?

Notes

Email address for gift card

____

That's my last question.

Information from all the practices and clinics will be combined to give us information about the issues faced by medical practices related to providing the HPV vaccine.

Do you have any questions? (Answer any questions at that time.)
Thank you very much for your time and cooperation.
